# Association of Kidney Function With NMR-Quantified Lipids, Lipoproteins, and Metabolic Measures in Mexican Adults

**DOI:** 10.1210/clinem/dgab497

**Published:** 2021-07-03

**Authors:** Diego Aguilar-Ramirez, Jesus Alegre-Díaz, William G Herrington, Natalie Staplin, Raúl Ramirez-Reyes, Louisa Gnatiuc, Michael Hill, Frederik Romer, Jason Torres, Eirini Trichia, Rachel Wade, Rory Collins, Jonathan R Emberson, Pablo Kuri-Morales, Roberto Tapia-Conyer

**Affiliations:** 1 Clinical Trial Service Unit & Epidemiological Studies Unit, Nuffield Department of Population Health, University of Oxford, Oxford, UK; 2 Faculty of Medicine, National Autonomous University of Mexico, Mexico City, Mexico; 3 MRC Population Health Research Unit, Nuffield Department of Population Health, University of Oxford, Oxford, UK

**Keywords:** kidney function, diabetes, Mexico, metabolic measures, nuclear magnetic resonance spectroscopy

## Abstract

**Context:**

Chronic kidney disease (CKD) and diabetes are associated with dyslipidemia, metabolic abnormalities, and atherosclerotic risk. Nuclear magnetic resonance (NMR) spectroscopy provides much more detail on lipoproteins than traditional assays.

**Methods:**

In about 38 000 participants from the Mexico City Prospective Study, aged 35 to 84 years and not using lipid-lowering medication, NMR spectroscopy quantified plasma concentrations of lipoprotein particles, their lipidic compositions, and other metabolic measures. Linear regression related low estimated glomerular filtration rate (eGFR; <60 mL/min/1.73 m^2^) to each NMR measure after adjustment for confounders and for multiplicity. Analyses were done separately for those with and without diabetes.

**Results:**

Among the 38 081 participants (mean age 52 years, 64% women), low eGFR was present for 4.8% (306/6403) of those with diabetes and 1.2% (365/31 678) of those without diabetes. Among both those with and without diabetes, low eGFR was significantly associated with higher levels of 58 NMR measures, including apolipoprotein B (Apo-B), the particle numbers of most Apo-B containing lipoproteins, the cholesterol and triglycerides carried in these lipoproteins, several fatty acids, total cholines and phosphatidylcholine, citrate, glutamine, phenylalanine, β-OH-butyrate, and the inflammatory measure glycoprotein-A, and significantly lower levels of 13 NMR measures, including medium and small high-density lipoprotein particle measures, very low-density lipoprotein particle size, the ratio of saturated:total fatty acids, valine, tyrosine, and aceto-acetate.

**Conclusions:**

In this Mexican population with high levels of adiposity and diabetes, low kidney function was associated with widespread alterations in lipidic and metabolic profiles, both in those with and without diabetes. These alterations may help explain the higher atherosclerotic risk experienced by people with CKD.

Chronic kidney disease (CKD) is a global public health priority with an age-standardized prevalence in adults of about 9% (1-3). It exhibits strong associations with risk of death from cardiovascular causes (4) including both atherosclerotic causes and structural heart disease (5). In observational studies, each 30% reduction in kidney function is associated with about a 30% increase in risk of major atherosclerotic cardiovascular diseases (6). Risk of myocardial infarction among people with mild-to-moderate CKD is similar to that in people with established coronary artery disease or diabetes and exceeds the risk in such individuals when estimated glomerular filtration rate (eGFR) falls below 45 mL/min/1.73 m^2^ (7). The coexistence of both CKD and diabetes is common (8), and the associations of each with cardiovascular disease are broadly independent (7,9). As such, people with both CKD and diabetes are at particularly high cardiovascular risk (9).

CKD is associated with a typical pattern of dyslipidemia, which is similar to that observed in people with diabetes (10,11). It is characterized by high plasma triglycerides, varying changes in low-density lipoprotein (LDL) cholesterol concentration (12), and lower high-density lipoprotein (HDL) cholesterol. This represents an underlying redistribution of cholesterol across lipoprotein subclasses resulting in increased triglyceride-rich very low density lipoprotein (VLDL) and intermediate density lipoprotein (IDL) particles, an increased proportion of small oxidized LDL particles, and increased apolipoprotein-B (Apo-B) (13, 14). CKD-associated dyslipidemia may be a key mediator of the excess cardiovascular risk experienced by people with CKD (12,15-17).

Previous studies investigating the relationship between eGFR and lipids have relied upon traditional blood assays and have been largely restricted to populations with advanced kidney disease or on dialysis (13,18,19). Nuclear magnetic resonance (NMR) spectroscopy offers detailed characterization of circulating plasma lipids, including lipoprotein concentrations by subclasses and information about lipidic composition, as well as characterizing other traits (Fig. 1). High-throughput NMR platforms have been developed for use in large-scale epidemiological studies (20-23), but current studies investigating associations between eGFR and NMR-quantified lipid and metabolic measures (referred to as “NMR measures” throughout this article) have been limited by their size (with the largest including about 6000 participants from 4 combined cohorts) (24) and were unable to explore associations comparatively in people with and without diabetes (24,25).

**Figure 1.**
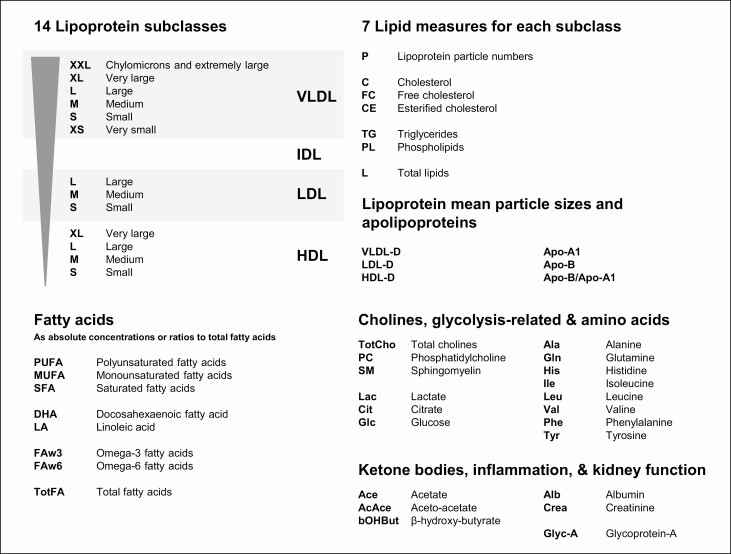
**Plasma lipid and metabolic measures quantified by nuclear magnetic resonance spectroscopy.** Abbreviations: Apo-A1, apolipoprotein A1; Apo B, apolipoprotein B; HDL, high density lipoprotein; HDL-D, HDL particle diameter; IDL, intermediate density lipoprotein; LDL, low density lipoprotein; LDL-D, LDL particle diameter; VLDL, very low density lipoprotein; VLDL-D, VLDL particle diameter. *Per the high-throughput NMR-metabolomics platform developed by Nightingale Health Ltd (21).

This paper describes the associations between reduced kidney function and NMR measures among about 38 000 individuals from the Mexico City Prospective Study, which recruited participants between 1998 and 2004 (26,27), when obesity and diabetes were already very common in Mexico, but use of lipid modifying therapies was not.

## Materials and Methods

### Recruitment and Baseline Assessment

From 1998 to 2004, 52 644 men and 107 111 women aged 35 years or older from 2 districts of Mexico City (Coyoacan and Iztapalapa) were visited in their homes and agreed to enroll in a prospective study (28). Trained nurses recorded sociodemographic and lifestyle factors, current medications, and medical history. Blood pressure, weight, height, waist circumference, and hip circumference were measured and a 10 mL blood sample was collected. Ethics approval was granted by the Mexican Ministry of Health, the Mexican National Council for Science and Technology, and the University of Oxford. All participants provided written informed consent.

### Blood Sample Handling, Storage, and Assays

Blood samples were transported to the central laboratory at 4°C to 10°C, refrigerated overnight at 4°C, and then separated the next morning. Plasma and buffy coat samples were briefly stored locally at −80°C, then transported on dry ice to Oxford (UK) for long-term storage over liquid nitrogen at −150°C. Such storage conditions ensure stability of blood lipid and other measures between collection and measurement (29) (and there was no association between storage time and eGFR in the current study). Glycosylated hemoglobin (HbA1c) was measured for all participants from the buffy coat sample using validated high-performance liquid chromatography methods (30) on HA-8180 analyzers (Arkray, Inc.) with calibrators traceable to International Federation of Clinical Chemistry standards (31). Between September 2018 and October 2019, a subset of 40 349 baseline plasma samples were subaliquoted and analyzed by NMR spectroscopy. The majority of samples were analyzed at Nightingale Health Ltd (Kuopio, Finland) with the remainder analyzed with the same protocol validated for use at the Clinical Trial Service Unit’s (CTSU) Wolfson laboratory (Oxford, UK). The Nightingale Health Ltd high-throughput targeted NMR metabolomics platform (21) generates spectra from which 228 NMR measures are quantified as absolute concentrations or ratios (Fig. 1). In addition, a random sample of 1000 baseline plasma samples were analyzed for standardized clinical chemistry measurements of total cholesterol, LDL cholesterol, HDL cholesterol, triglycerides, Apo-B, Apo-A1, and creatinine at the CTSU Wolfson laboratory. As reported previously (21), the automated NMR platform has multiple and standardized quality control checkpoints at both plate and batch level. In addition, an interlaboratory comparison between Nightingale Health Ltd and CTSU’s Wolfson Laboratory was performed (using multiple sets of samples run in both laboratories) with good agreement observed.

### Statistical Analysis

Analyses were limited to those aged 35 to 84 years not taking lipid-lowering drugs when recruited. Those with missing or implausible values for NMR-measured creatinine, HbA1c, or relevant confounders (see following discussion) were also excluded. Although serum and plasma creatinine have been validated for use on both the Nightingale Health Ltd NMR-platform and the clinical chemistry used in this study, we adjusted the original NMR-measured creatinine values (in μmol/L) through the formula *y* = 3.56 + 1.05*x*, derived from the linear relationship between NMR-measured creatinine and isotope dilution mass spectrometry–traceable clinical chemistry-measured creatinine in the subset with both [an approach that is acceptable (9) when using creatinine assays not traceable to isotope dilution mass spectrometry (32)]. (Analyses without this adjustment gave similar results.) Each participant’s eGFR was then calculated using the Chronic Kidney Disease Epidemiology Collaboration equation (33) categorizing individuals as nonblack (consistent with previous Hispanic-US and Mexican population studies) (34,35). Participants were then categorized into 2 groups based on the clinical cutoff for CKD stage G3 (eGFR ≥ 60 *vs* < 60 mL/min/1.73 m^2^). Results across wider categories of eGFR are provided in Figure 2 of the supplemental material (36).

The 138 NMR measures (other than creatinine) were log-transformed and then normalized (ie, subtraction of the mean and division by the SD), with values lower than the detection limit assigned the lower detection limit. Linear regression was then used to relate low eGFR (ie, eGFR < 60 mL/min/1.73 m^2^) to each NMR measure. Analyses were done separately among those with and without diabetes [defined as either previously diagnosed (ie, self-reported previous medical diagnosis or the use of any anti-diabetic medication) or undiagnosed (no previous diagnosis but HbA1c ≥6.5%)]. Regression models were adjusted for age (in five 10-year categories), sex, highest attained level of education (university or college, high school, primary school, other), district of residence (2 districts), smoking status (never, former, occasional, <10 cigarettes per day, ≥10 cigarettes per day), fasting duration (4 equally sized groups), and NMR batch number (8 groups). Sensitivity analyses further adjusted for body-mass index and waist-to-hip ratio, and, in those with diabetes only, NMR-measured albumin or a combination of HbA1c level and insulin use.

Regression estimates are shown with 95% CIs. To account for the large number of assessments (ie, multiplicity), the false discovery rate was controlled at 5% using the Benjamini-Hochberg method (37). Associations are considered significant if the false discovery rate–adjusted *P*-value was < 0.05. Analyses were performed with SAS, version 9.4 (SAS Institute) and R, version 4.0.2 (www.r-project.org/). Circular plots were made with the R package “RCircos” (38).

## Results

Of the 159 755 recruited participants, 148 661 (93%) were 35 to 84 years of age, were not taking a lipid-lowering drug, and had complete data. Of these, 38 081 (26%) had NMR measurements available including a valid measurement of creatinine [levels of missing data shown in Supplementary Table 1 in (36)]. The 38 081 participants included 6403 (17%) with and 31 678 (83%) without diabetes (Table 1).

**Table 1.** Characteristics of adults aged 35 to 84 years by eGFR categories in individuals with and without diabetes at recruitment

	Individuals with diabetes (*n* = 6043)			Individuals without diabetes (*n* = 31 678)			
	eGFR ≥ 60 mL/min/1.73 m^2^ (*n* = 6097)	eGFR < 60 mL/min/1.73 m^2^ (*n* = 306)	All with diabetes (*n* = 6043)	eGFR ≥ 60 mL/min/1.73 m^2^ (*n* = 31 313)	eGFR < 60 mL/min/1.73 m^2^ (*n* = 365)	All without diabetes (n = 31 678)	All participants (*n* = 38 081)
eGFR, mL/min/1.73 m^2^	100 (14)	38 (16)	97 (19)	102 (13)	46 (15)	102 (14)	101 (15)
Age	58 (11)	65 (9)	58 (11)	50 (12)	69 (12)	50 (12)	52 (12)
Male sex	2214 (36%)	101 (33%)	2315 (36%)	11 334 (36%)	121 (33%)	11 455 (36%)	13 770 (36%)
Resident of Coyoacán	5398 (89%)	263 (86%)	5661 (88%)	28 338 (90%)	303 (83%)	28 641 (90%)	34 302 (90%)
University/college educated	457 (7%)	8 (3%)	465 (7%)	5225 (17%)	26 (7%)	5251 (17%)	5716 (15%)
Current smoker	1472 (24%)	40 (13%)	1512 (24%)	9381 (30%)	48 (13%)	9429 (30%)	10 941 (29%)
Anthropometry, blood pressure, and HbA1c							
Body-mass index, kg/m^2^	29.3 (5.2)	27.7 (4.7)	29.2 (5.2)	28.6 (4.8)	28.5 (4.8)	28.6 (4.8)	28.7 (4.8)
Waist-hip ratio	0.93 (0.07)	0.93 (0.08)	0.93 (0.07)	0.90 (0.08)	0.94 (0.08)	0.90 (0.08)	0.90 (0.08)
SBP, mmHg	136 (18)	150 (25)	136 (19)	128 (16)	141 (22)	128 (17)	129 (17)
HbA1c (%)	8.6 (6.8-10.7)	7.2 (6.3-9.1)	8.5 (6.8-10.7)	5.4 (5.1-5.5)	5.4 (5.2-5.6)	5.4 (5.1-5.5)	5.4 (5.2-5.8)
Self-reported comorbidities							
Cardiovascular disease	289 (5%)	33 (11%)	322 (5%)	721 (2%)	29 (8%)	750 (2%)	1072 (3%)
Chronic kidney disease	76 (1%)	39 (13%)	115 (2%)	281 (1%)	29 (8%)	310 (1%)	425 (1%)

Values are mean (SD), n (%), or median (interquartile range). The glomerular filtration rate was estimated using the Chronic Kidney Disease Epidemiology Collaboration equation and NMR-measured creatinine that was recalibrated to a reference creatinine measured by isotope dilution mass spectrometry available in a subset of participants. Pearson’s correlation coefficients for NMR- and IDMS-measured creatinine were r = 0.89 (n = 282) [see Supplementary Figure 1 in (36)]. Mean (SD) NMR-measured and recalibrated creatinine values were (following the order of the columns): 63 (12), 160 (174), 64 (25), 606 (13), 183 (137), 66 (42), and 64 (28) umol/L. Abbreviations: eGFR, estimated glomerular filtration rate; HbA1c, glycosylated haemoglobin; SBP, systolic blood pressure.

Among the 38 081 participants, mean eGFR was 101 (SD 15) mL/min/1.73 m^2^, mean age was 52 (SD 12) years, and just over one third were male. Compared to those without diabetes, those with diabetes were older (58 *vs* 50 years) and had lower mean eGFR (97 *vs* 102 mL/min/1.73 m^2^). Three hundred eleven participants (5%) with diabetes were taking insulin. Low kidney function (ie, eGFR < 60 mL/min/1.73 m^2^) was present in 306 (4.8%) participants with diabetes and 365 (1.2%) participants without diabetes. Ninety-one (1.4%) participants with diabetes and 52 (0.2%) participants without diabetes had eGFR < 30 mL/min/1.73 m^2^ and 756 (12%) and 4635 (15%), respectively, had eGFR > 120 mL/min/1.73 m^2^ [see Supplementary Table 2 in (36)]. Participants with low eGFR were older, less likely to have attended college or university, and less likely to be current smokers. Among those with both NMR and standard clinical chemistry measurements, the correlation between the 2 estimation methods was good to high for all markers [see Supplementary Figure 1 in (36)]. Supplementary Table 3 in (36) provides the mean concentrations of the 138 NMR measures.

### Association Between Low Kidney Function and NMR Measures

**Figure 2.**
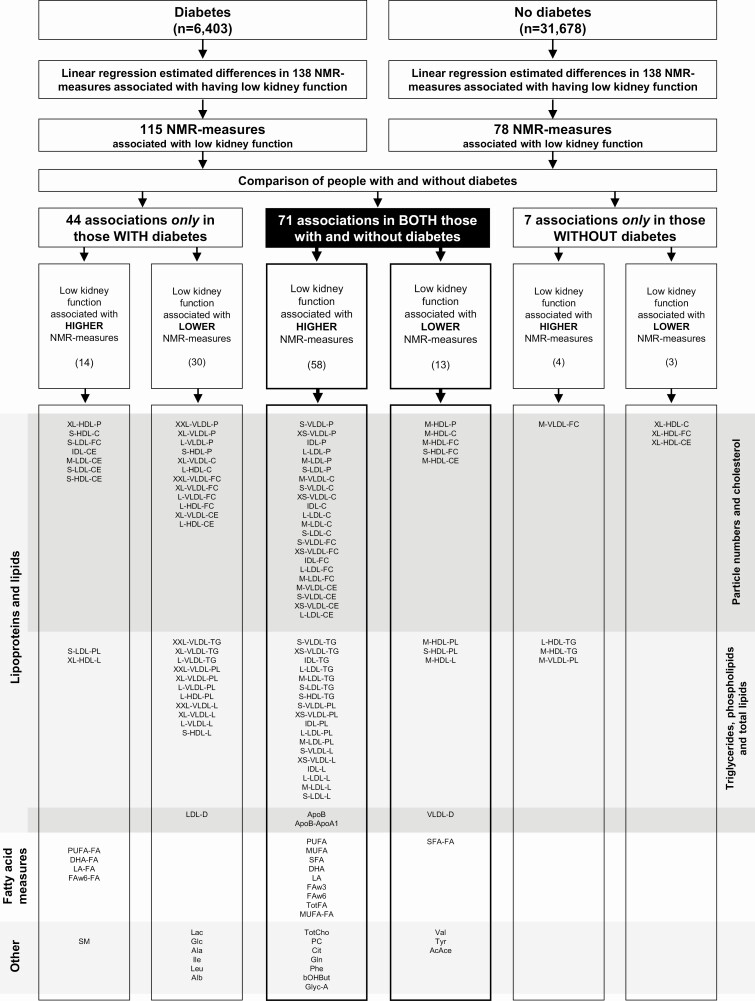
**Workflow for identifying associations of low kidney function (eGFR < 60 mL/min/1.73 m^2^) with NMR measures and the comparison between people with and without diabetes.** Associations between low kidney function and NMR measures were assessed separately in people with and without diabetes using linear regression models adjusted for age, sex, district of residence, educational level, smoking, and fasting duration. A false discovery rate–adjusted *P*-value < 0.05 was considered as evidence against the null hypothesis. NMR measures nomenclature is defined in Figure 1.

**Figure 3.**
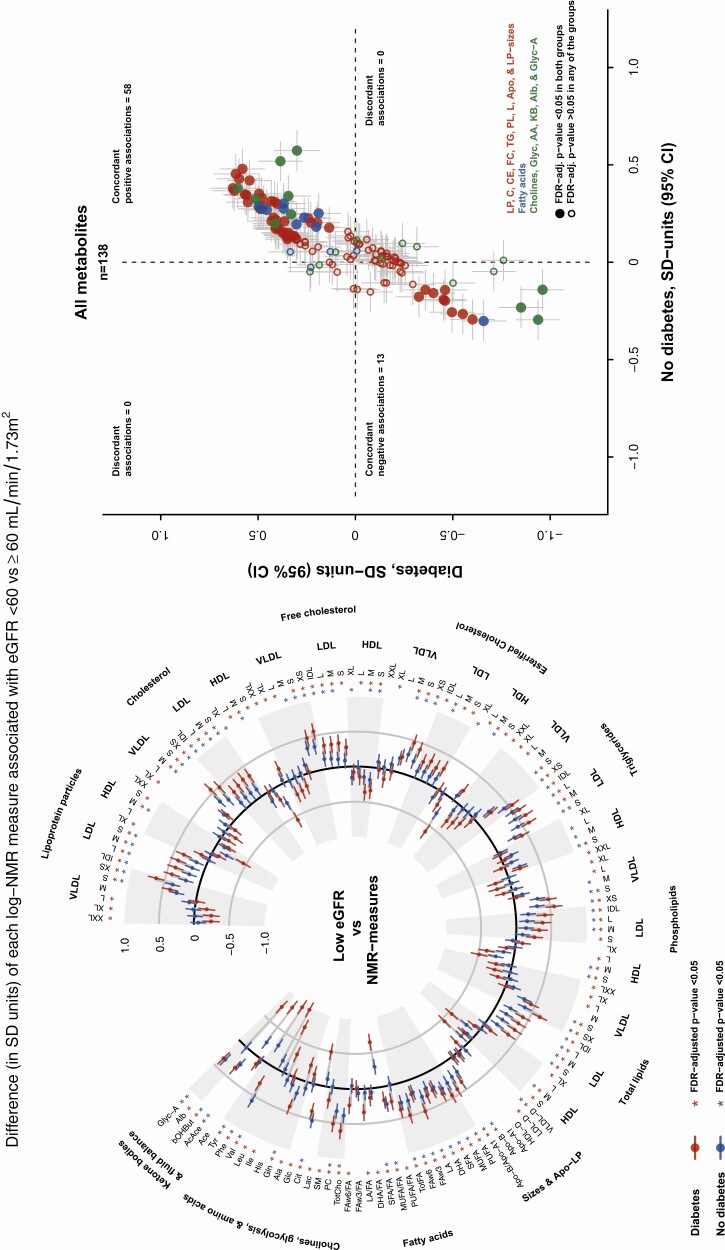
**Comparison of associations of low kidney function (eGFR < 60 mL/min/1.73 m^2^) with NMR-quantified lipid and metabolic measures between adults with and without diabetes.** Linear regression models are adjusted for age, sex, educational level, district of residence, smoking, fasting duration, and batch number. Point estimates for each association are available in Supplementary Table 4 in (36). NMR measures nomenclature is defined in Figure 1. Abbreviations: eGFR, estimated glomerular filtration rate; FDR, false discovery rate. NMR, nuclear magnetic resonance.

Low eGFR was significantly associated with 115 NMR measures in participants with diabetes and with 78 NMR measures in participants without diabetes (Figs. 2 and 3). Of these associations, 44 were unique to those with diabetes, 7 were unique to those without diabetes, and 71 were shared for both those with and without diabetes. All of the 71 significant associations that were seen in both those with and without diabetes showed concordant directionality in the 2 populations [Fig. 3; also see Supplementary Table 4 in (36)].

#### Concordant associations with lipoproteins, lipids, and apolipoproteins

**Figure 4.**
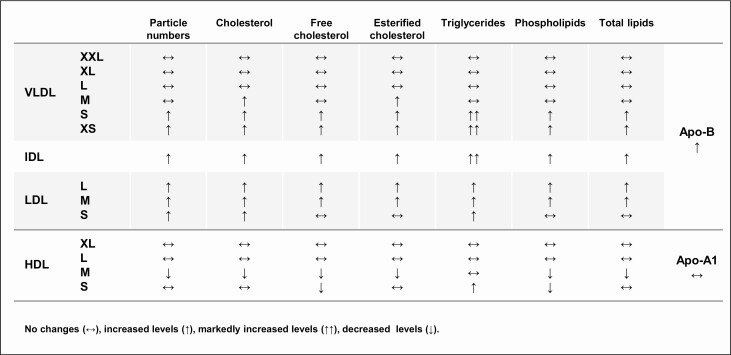
**Qualitative changes in lipoprotein and lipid measures associated with low kidney function shared among people with and without diabetes.** NMR measures nomenclature defined in Figure 1.

Low eGFR was associated with higher levels of Apo-B and the particle numbers of most Apo-B containing lipoproteins (from small and very small VLDL to IDL and all LDL) as well as with the cholesterol, triglycerides, and phospholipids within those lipoproteins. The strongest positive association among those with diabetes was for very small VLDL particles, while the strongest positive association among those without diabetes was for triglycerides in IDL. For very small VLDL particles, low eGFR was associated with 0.63 higher SD units in those with diabetes and 0.38 higher SD units in those without diabetes, while for triglycerides in IDL low eGFR was associated with 0.58 higher SD units in those with diabetes and 0.48 higher SD units in those without diabetes. The Apo-B to apolipoprotein-AI (Apo-AI) ratio was also higher in those with low eGFR (Figs. 2-4).

Contrastingly, low eGFR was associated with lower levels of medium HDL measures including the particle numbers, cholesterol, and phospholipids. The strongest inverse association was for esterified cholesterol in medium HDL, for which low eGFR was associated with 0.60 lower (95% CI 0.49-0.72) SD units among those with diabetes and 0.29 lower (95% CI 0.19-0.40) SD units in those without diabetes. The average size of VLDL (VLDL particle diameter) was also lower in those with low eGFR (Figs. 2-4).

#### Concordant associations with fatty acids

Low eGFR was associated with higher levels of all fatty acid measures (as absolute concentrations) and higher levels of the ratio of monounsaturated fatty acids to total fatty acids. The strongest positive association among those with diabetes was for linoleic acid, while the strongest positive association among those without diabetes was for mono-unsaturated fatty acids. For linoleic acid, the associations in those with *vs* without diabetes were 0.50 higher (95% CI 0.38-0.61) SD units and 0.29 higher (95% CI 0.18-0.39) SD units, respectively, while for mono-unsaturated fatty acids they were 0.30 higher (95% CI 0.20, 0.40) SD units and 0.30 higher (95% CI 0.20, 0.40) SD units, respectively. The ratio of saturated fatty acids to total fatty acids was the only fatty acid–based measurement for which low eGFR was associated with a lower level (Figs. 2 and 3).

#### Concordant associations with other NMR measures

Low eGFR was associated with higher levels of total cholines, phosphatidylcholine, citrate, glutamine phenylalanine, beta-hydroxy-butyrate, and the inflammatory measure glycoprotein-A. By contrast, low eGFR was associated with lower levels of valine, tyrosine, and aceto-acetate (Figs. 2 and 3).

#### Associations only in those with diabetes or in those without diabetes

There were 44 NMR measure that were associated with low eGFR only in those with diabetes (Figs. 2 and 3). In this group, low eGFR was associated with higher levels of 14 measures, including the free and esterified cholesterol and phospholipids in small LDL and esterified cholesterol IDL, the concentrations of very large HDL, and the cholesterol in small HDL, as well as with 4 fatty acid measures and with sphingomyelin. Low eGFR was also associated with lower levels of 30 other measures including most measures of the 3 largest sizes of VLDL (particles, cholesterol, triglycerides, and phospholipids), with lower LDL mean particle size, and with lower levels of the particles, cholesterol, and phospholipids in large HDL, as well as lactate, glucose, alanine, isoleucine, leucine, and albumin. Uniquely among those without diabetes, 7 NMR measures were associated with low eGFR, including higher levels of medium VLDL free cholesterol and large and medium HDL triglycerides and with lower levels of cholesterol in very large HDL (Figs. 2 and 3).

#### Subsidiary and sensitivity analyses

Supplementary Figure 2 in (36) shows the associations between levels of eGFR (cutoffs > 120, 90-120, 60-89, <60 mL/min/1.73 m^2^_,_ those with 90 to 120 mL/min/1.73 m^2^ were used as reference group) and NMR measures in people with and without diabetes. Similar patterns of associations were found, with a large number of the associations showing an approximately linear relationship between levels of eGFR and the log NMR measure. The main analyses of the association between low eGFR and the NMR measures were largely unchanged when further adjusted for body mass index and waist-hip ratio [see Supplementary Figure 3 in (36)]. Among those with diabetes, results were also little affected by further adjustment for albumin [see Supplementary Figure 4 in (36)] or further adjustment for HbA1c and use of insulin [see Supplementary Figure 3 in (36)].

## Discussion

This study shows that an eGFR consistent with early stage CKD is associated with widespread differences in NMR-quantified circulating plasma lipids, lipoproteins, and other measures, many of which are not characterized by traditional blood lipid panels. The majority of these associations are shared in those with and without diabetes. Low kidney function was associated with higher levels of the particle numbers of most Apo-B containing lipoproteins as well as the subfractions of cholesterol, triglycerides, and phospholipids related to these lipoproteins (and therefore also with higher Apo-B). Apo-B differences associated with low kidney function tended to be larger among those with than without diabetes. Concentrations of several fatty acids and other NMR measures including the inflammation trait glycoprotein-A were also higher among those with low kidney function.

Studies based on traditional lipid panels have found low kidney function associates with higher absolute concentrations of triglycerides and, depending on the CKD stage, with varying levels of Apo-B, total cholesterol, LDL cholesterol, and non-HDL cholesterol (13,14). NMR profiling shows that this dyslipidemic pattern is specific to certain lipoprotein subclasses (ie, small and very small VLDL, IDL, and all LDL), and notably, low kidney function was associated with an increased number of these lipoproteins (ie, it was not limited to particle’s lipidic contents). As each VLDL, IDL, and LDL have exactly 1 Apo-B on their surface, the overall numbers of circulating Apo-B were higher in those with low kidney function with or without diabetes, respectively (Fig. 4).

There have been conflicting reports on the association between kidney function and Apo-B. Previous population-based studies with NMR measures either did not find an association between eGFR and Apo-B (24) or identified it only among people with diabetes (25). Small case-control studies from hemodialysis populations have shown no changes or even lower levels of Apo-B in those at these advanced stages of CKD (compared to healthy individuals) (39,40). In the present study, low eGFR was clearly associated with higher levels of Apo-B. Furthermore, when eGFR was modeled as categories, individuals with an eGFR < 60 and between 60 and 89 were associated with higher levels of Apo-B compared to the reference group of those with an eGFR between 90 and 120 mL/min/1.73 m^2^ [see Supplementary Figure 3 in (36)]. Although the present study included a relatively low number of participants with eGFR levels consistent with advanced CKD (ie, eGFR < 30 mL/min/1.73 m^2^), increased levels of Apo-B were clearly present even at the earliest stages of CKD and may contribute importantly to progressively increased atherosclerotic disease risk in people with progressively low eGFR, a risk that is reduced by statin-based therapies (12,15).

The average diameter of triglyceride-rich lipoproteins is <40 nm (as measured by NMR) (21), which is sufficiently small to infiltrate endothelia and deposit within the tunica intima and plaques. The absolute concentrations of these smaller VLDL were higher at low kidney function than the larger VLDL particles, irrespective of diabetes [see Supplementary Table 3 in (36)] and may contribute to atherosclerotic risk in CKD, as has been found in other populations (20). Such lipid changes are difficult to identify on traditional lipid panels, as increases in these lipoproteins are split between total triglycerides, Apo-B, and non-HDL cholesterol measures.

Based on traditional blood lipid measurements, decreased Apo-AI concentration in people with CKD results in low HDL cholesterol concentration (13). Under normal physiological conditions, Apo-AI mediates the esterification of cholesterol in HDL particles, increasing the absolute amount of cholesterol transported in enlarged HDL particles (ie, decreased Apo-AI relates to impaired HDL function) (13). In our analyses, low kidney function was not associated with Apo-AI concentration, and the associations with lower levels of HDL measures were restricted to those in medium-sized HDL (cholesterol and particle numbers). Large-scale randomized trial evidence (41-44) and genetic studies (45-46) have shown little effect of HDL cholesterol on coronary heart disease risk. Taken together, these data suggest disturbances in HDL at low levels of kidney function are probably less clinically relevant to atherosclerotic risk in CKD than the changes to Apo-B containing and triglyceride-rich lipoproteins.

Our analyses extend those of previous studies. Low kidney function has previously been associated with fatty acids (25), phenylalanine, and the branch-chain amino acids valine, isoleucine, and leucine (24,25). These traits have been linked to increased adiposity and insulin resistance and the development of diabetes (47). Increased levels of the inflammatory trait glycoprotein-A (which was also observed at low kidney function) has been associated with high cardiovascular risk (20,48). As randomized evidence (49) has provided support for a causal nature of (at least some) inflammation pathways in the development of cardiovascular disease, a randomized clinical trial targeting inflammation reduction within the context of CKD (even at early stages of CKD) would be of value.

To our knowledge, this is the largest study to assess associations between kidney function and a detailed panel of NMR measures in people with and without diabetes from the same population. Previous studies have been limited to populations with diabetes (25) or pooled from different populations (24). The use of a clinically relevant binary categorization of the exposure generated clear associations, and our explorations of the shape of these relationships showed a linear relationship between categories of eGFR and many NMR measures. A limitation of this NMR-metabolomics platform is that it does not measure lipoprotein (a) [Lp(a)] or the oxidization of LDL particles, both of which have positive associations with CKD and cardiovascular risk (14). The association of low eGFR with Lp(a) is, however, indirectly included within its association with Apo-B, as each Lp(a) contains 1 Apo-B. Other limitations are that analyses are based on a single measure of eGFR rather than at least 2 spaced by >3 months, and there were low numbers of participants with the most advanced stages of CKD (ie, eGFR < 30 mL/min/1.73 m^2^). These 2 limitations mean associations are likely to be underestimates of the full effect of CKD on NMR measures. Additionally, we have not been able to directly consider the impact of albuminuria on NMR measures. Nephrotic range proteinuria is associated with hyperlipidemia and lower levels of Apo-AI (13), but associations of nonnephrotic albuminuria and lipids remains relatively unexplored (25). Lastly, it may not be appropriate to infer causality for many of the associations identified in this report. Genetic data on the whole cohort will soon exist, however, which will allow such assessments to be made through Mendelian randomization approaches.

In summary, the metabolic profile of CKD appears to be characterized by multiple effects on lipoproteins and their lipidic content, with substantially increased absolute numbers of, and the lipids within, atherosclerotic Apo-B containing lipoproteins as well as Apo-B, and increased concentrations of the smallest VLDL particles. The effects of low kidney function on Apo-B were somewhat stronger in those with diabetes. A better understanding of these differences should help direct future research into how to modify atherosclerotic risk among people with reduced kidney function, who remain at high residual risk despite intensive LDL-lowering therapy.

## Data Availability

We welcome requests from researchers who wish to access data from the Mexico City Prospective Study. If you are interested in obtaining data from the study for research purposes or in collaborating with us on a specific research proposal, please visit our study website [https://www.ctsu.ox.ac.uk/research/prospective-blood-based-study-of-150-000-individuals-in-mexico] where you can download our Data and Sample Access Policy in either English or Spanish.
